# Medicinal Properties of *Lilium candidum* L. and Its Phytochemicals

**DOI:** 10.3390/plants9080959

**Published:** 2020-07-29

**Authors:** Michele Zaccai, Ludmila Yarmolinsky, Boris Khalfin, Arie Budovsky, Jonathan Gorelick, Arik Dahan, Shimon Ben-Shabat

**Affiliations:** 1 Department of Life Sciences, Ben-Gurion University of the Negev, Beer-Sheva 8410501, Israel; mzaccai@bgu.ac.il; 2 Eastern R&D Center, Kiryat Arba 9010000, Israel; liorayarl@mail.com (L.Y.); boriskh83@gmail.com (B.K.); jonathangorelick@gmail.com (J.G.); 3 Faculty of Health Sciences, Ben-Gurion University of the Negev, Beer-Sheva 8410501, Israel; arikd@bgu.ac.il; 4 Research & Development Authority, Barzilai University Medical Center, Ashkelon 7830604, Israel; abudovsky@gmail.com

**Keywords:** *Lilium candidum*, phytochemicals, medicinal activities

## Abstract

*Lilium candidum* L., known as Madonna, meadow, or white lily, is a bulbous plant from the Liliaceae family, originating in the Middle East. *L. candidum* has been abundantly used in folk medicine since ancient times to relieve a variety of ailments, including age-related diseases, burns, ulcers, and coughs. The aim of this article is to investigate the anti-inflammatory and anti-diabetic activities of *L. candidum* extracts and its active phytochemicals. Some active volatile phytochemicals were identified using gas chromatography–mass spectrometry (GC-MS) analysis. Significant (*p* < 0.001) anti-diabetic properties of the extracts kaempferol, linalool, citronellal, and humulene were demonstrated by an elevation in glucose uptake by adipocytes. The significant (*p* < 0.01) effect of the plant extracts kaempferol, citronellal, and humulene on the secretion of pro-inflammatory cytokines interleukin 6 (IL-6) and interleukin 8 (IL-8) was demonstrated using enzyme-linked immunosorbent assay. Altogether, *L. candidum* and its rich collection of phytochemicals hold promising medicinal potential, and further investigations of its therapeutic prospects are encouraged.

## 1. Introduction

The prevalence of age-related diseases (ARDs) including cardiovascular diseases, cancer, type 2 diabetes, neurodegenerative diseases, and obesity is rapidly increasing worldwide [1]. Recent studies provide strong evidence suggesting an essential role of chronic inflammation in the pathogenesis of the above-mentioned ARDs [2,3,4]. In addition, genotoxic stress, which is a component of a wide variety of pathological conditions, not only causes extensive DNA damage but also activates pathways leading to chronic inflammation (the ERK, JNK, and p38 MAPK pathways) and transcription of pro-inflammatory cytokines (TNF-α, IL-1β, interleukin 6 (IL-6)), chemokines (interleukin 8 (IL-8)), adhesion molecules (VCAM-1, ICAM-1, P-, E-selectin), and other pro-inflammatory enzymes including iNOS and COX-2 [5]. This in turn leads to an elevated pro-inflammatory status that is likely to set the stage for increased vulnerability to many ARDs [6].

Unfortunately, current therapeutic agents have inadequate efficacy and many serious adverse effects in treating all kinds of ARDs [7]. All possible options should be considered in order to develop new drugs that are more effective. In fact, many medicinal plants are able to cope with inducers and/or consequences of stress such as thermal or oxidative insults, ionizing radiation, DNA damage, exposure to carcinogens, and inflammatory burden, which is one of the important determinants of survival and longevity [8,9].

*Lilium candidum* L. has been well known in folk medicine for a long time, not only in the plant’s native regions (Balkans, Middle East) but also in other parts of the world in which it was naturalized, such as various European countries, North Africa, and Mexico. In folk medicine worldwide, *L. candidum* is prominently associated with dermal conditions, cosmetics, and anti-inflammatory remedies [10,11,12]. A vast ethnopharmacological research study performed in the Campidano Valley and Urzulei district in Italy revealed many medicinal benefits of *L. candidum*. Among them were application of lily petals and a decoction of bulbs soaked in milk as pectoral poultices, application of petals soaked in spirit as a wound-healing remedy, and the use of oil prepared from flowers as a treatment for mastitis [13]. In addition, ethnopharmacological research in Lucca province in Italy demonstrated the use of *L. candidum* bulbs as an anti-viral agent to treat shingles (Herpes zoster), and its bulbs and flowers for treatment of skin and articular diseases [14]. *L. candidum* was also successfully used in anti-inflammatory and dermatological remedies in the Catalan district of the Eastern Pyrenees [14].

*L. candidum* L., commonly known as Madonna, meadow, or white lily, is a geophyte from the Liliaceae family growing in the wild in several countries of the Middle East. The origin of *L. candidum* is believed to be in Lebanon and Israel, as well as several parts of Greece [15,16,17], thus Israel represents the Southern border of *L. candidum* distribution and only few populations are found in the Carmel and the Galilee regions. It is considered an endangered species and as such the plants are protected [15,18]. A collection of wild *L. candidum* ecotypes from different locations exhibited genetic variation in morphologic and phenologic traits, as well as in phytotoxicity of their leaf extracts [18,19].

Effective delivery of herbal compounds and plant extracts is a very important issue, since drawbacks such as hydrophobicity, insolubility in water, high volatility, and instability pose a challenge [20,21]. The application of innovative drug delivery systems including phytosomes, nanoparticles, hydrogels, microspheres, transferosomes and ethosomes, self-nanoemulsifying drug delivery systems (SNEDDS), self-microemulsifying drug delivery systems (SMEDDS) and so on may improve the biopharmaceutical features of the delivered compounds [22,23].

Owing to its rare beauty, its fragrance, and its glorious symbolism, *L. candidum* appears to be a fascinating plant. It is therefore not surprising that Madonna lily has also been sought for therapeutic reasons. However, most *L. candidum* therapeutic properties known from folk tradition have not yet been investigated by scientific methods except anti-fungal [24], anti-cancer [25,26], and anti-viral [27] properties. Thus, the present study is aimed at identifying the presence of selected volatile compounds of the plant, assessing the medicinal potential of all known compounds using bioinformatics, and investigating anti-inflammatory and anti-diabetic activities of *L. candidum* extract and its active phytochemicals.

## 2. Results

**Figure 1:**
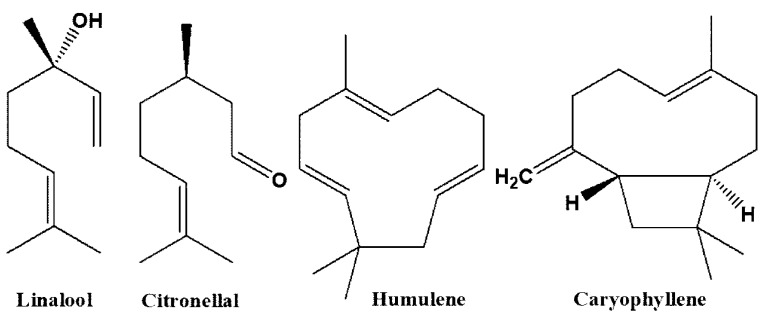
Chemical structures of compounds isolated from *L. candidum*. A computerized GC-MS (GC-6890N) equipped with a mass selective (MS)—5973 network (electron ionization 70 eV) detector from Agilent Technologies (Santa Clara, CA, USA) was used. Component recognition was performed by a comparison of the retention time index (RI) of the components to commercial standards and the samples’ mass spectrum with GC-MS libraries: Adams 2001, NIST 98, and QuadLib 1607.

We identified the presence of many novel volatile compounds using gas chromatography–mass spectrometry (GC-MS) analysis, which, to the best of our knowledge, were not mentioned in the literature. Among those, we found linalool, citronellal, caryophyllene, humulene (Figure 1), and neridiol (not represented), which were isolated and identified not only from *L. candidum* but from other plants as well.

Table 1 provides a list of these phytochemicals and their medicinal properties.

While it is clear that *L. candidum* possesses many valuable compounds with considerable therapeutic potential, to the best of our knowledge, no bioinformatical research has been carried out to assess their medicinal potential. In order to close this gap, we analyzed the potential human therapeutic targets (proteins and other biomolecules) of the above-mentioned compounds, using the STITCH database (http://stitch.embl.de/) [63,64,65].

**Figure 2:**
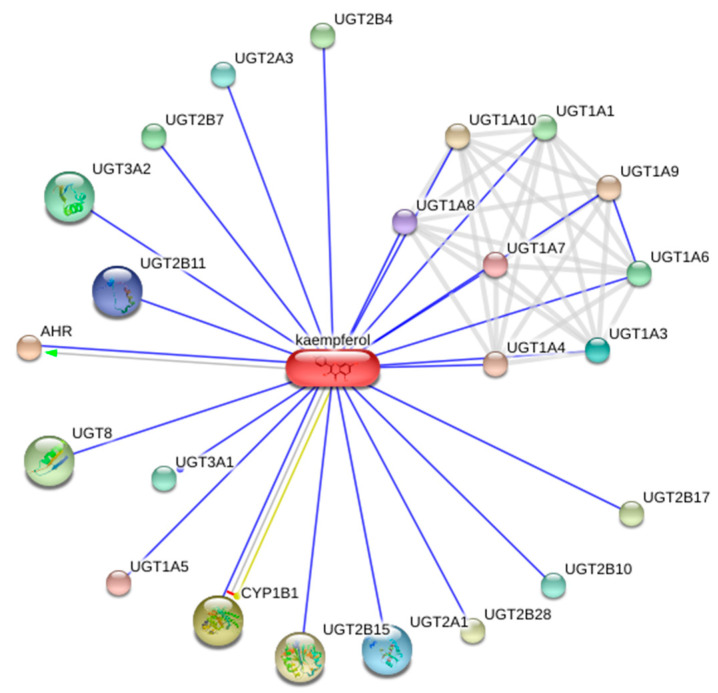
Direct protein–protein and protein–compound interactions between human targets of kaempferol. This figure was constructed using the default settings of the STITCH database (http://stitch.embl.de/). The uridine 5’-diphospho-glucuronosyltransferase (UGT), aryl hydrocarbon receptor (AHR), and cytochrome P450 family 1 subfamily B member 1 (CYP1B1) proteins groups are dominant.

In the case of kaempferol, a significant number of interacting protein targets were found to be associated with three large groups: UGT (uridine 5’-diphospho-glucuronosyltransferase), AHR (aryl hydrocarbon receptor), and CYP1B1 (cytochrome P450 family 1 subfamily B member 1) (Figure 2). The majority of the targets are connected to the UGT enzymes, which participate in cellular detoxification in different tissues of the digestive system [66]. Their involvement in various cancer-associated processes is also well-known [66,67]. AHR is a ligand-activated transcription factor, which may interact with different pathways regulating cellular homeostasis including cellular regeneration in the context of aging and diseases [68]. In addition, AHR regulates CYP1B1 [69], which belongs to the cytochrome P450 superfamily of enzymes. Cytochrome P450 plays an important role in cellular detoxification and in the formation of reactive intermediates of thousands of chemicals [70]. Thus, the medicinal effects of kaempferol are apparently mediated by its direct involvement in many pathological processes.

**Figure 3:**
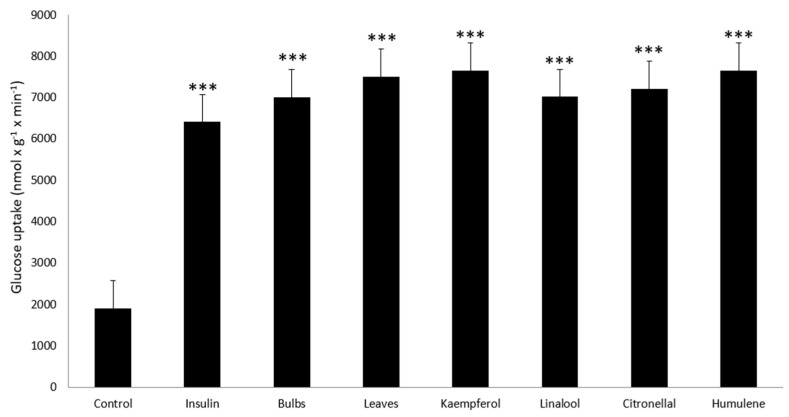
Anti-diabetic activity of *L. candidum* extracts and compounds. The glucose uptake of the 3T3 adipocytes was determined. Adipocytes were treated with ethanol extracts of *L. candidum* and with the compounds detected in those extracts. Negative control consisted of untreated adipocytes; insulin was used as a positive control. Data from three independent experiments are shown (mean ± SD). *** *p* < 0.001.

It is well acknowledged that diabetes mellitus (DM) pathogenesis is linked to oxidative stress [71]. Although many phytochemicals from *L. candidum* have anti-oxidant properties (Table 1), to the best of our knowledge, no research on its anti-diabetic properties has been performed so far. Thus, we investigated the anti-diabetic activity of bulbs and leaves of *L. candidum*. For this purpose, we treated adipocytes with an ethanolic extract from *L. candidum* bulbs and leaves, while the glucose uptake of the adipocytes was estimated after these treatments, as previously. *L. candidum* extracts increased glucose uptake in 3T3-L1A cells better than insulin. Importantly, the anti-diabetic activity of leaf extracts was higher than those from bulb extracts and of insulin, which was used as a positive control (Figure 3). The difference between negative control (untreated adipocytes) and cells that were treated with extracts was highly significant (*p* < 0.001). All known phytochemicals of *L. candidum* were tested in this experiment. As seen in Figure 3, kaempferol, linalool, citronellal, and humulene significantly increased glucose uptake in 3T3-L1 adipocytes (*p* < 0.001).

**Figure 4:**
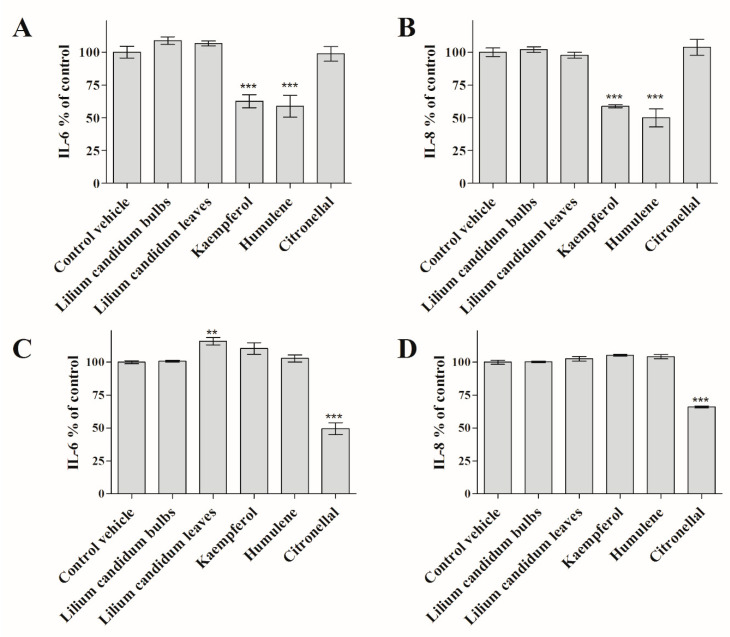
Effect of *L. candidum* ethanolic extracts and compounds on the release of pro-inflammatory cytokines interleukin 6 (IL-6) (**A**,**C**) and interleukin 8 (IL-8) (**B**,**D**) from human pulmonary fibroblasts (HPFs) (**A**,**B**) or human dermal fibroblasts (HDFs) (**C**,**D**). The ethanolic extracts were diluted in medium to a final ethanol concentration of 0.1%. Cells treated only with 0.1% ethanol (the vehicle) were used as controls to exclude the effect of ethanol on the cells. Data from three independent experiments are shown (mean ± SE). ** *p* < 0.01, *** *p* < 0.001.

Elevated levels of circulating inflammatory mediators including cytokines and chemokines are hallmarks of chronic inflammation and progression of metabolic diseases [2]. The extracts and their phytochemicals (identified by us and known) were investigated from the perspective of chronic inflammation. As seen in Figure 4, plant extracts, kaempferol, citronellal, and humulene significantly decreased secretion of IL-6 and IL-8 cytokines by senescent human pulmonary fibroblasts (HPFs) and human dermal fibroblasts (HDFs) as measured by enzyme-linked immunosorbent assay (ELISA) (*p* < 0.01).

## 3. Discussion

Taking into account the therapeutic properties of *L. candidum* known from folk tradition, we expected to discover anti-inflammatory and anti-diabetic activities in *L. candidum* extracts. It is important to emphasize here that senescent cells (HPFs and HDFs) are one of the most widely used models for studying inflammatory processes. Accumulation of senescent cells in an organism leads to disruption of tissues and cellular structure and function [72]. The phenomenon of cellular senescence has been demonstrated to play a causal role in driving ageing [72] and chronic diseases [7,9,72]. Namely, the above-mentioned cells are accepted models [7,9] used to investigate the effect of plant extracts and pure compounds on inflammation.

Our hypothesis was confirmed, as the plant extracts indeed possess anti-inflammatory and anti-diabetic properties (Figure 3 and Figure 4). We also identified selected phytochemicals behind these properties: anti-diabetic properties were associated with kaempferol, linalool, citronellal, and humulene (Figure 3). In turn, kaempferol, citronellal, and humulene had a significant impact on the secretion of pro-inflammatory cytokines IL-6 and IL-8 (*p* < 0.01), (Figure 4). It is known that the above-mentioned cytokines are directly connected to delay in the wound healing process and are known to be secreted by HPFs [9,73]. Thus, our results might provide an explanation of the effect of *L. candidum* extract on wound healing, as described in ethnopharmacological publications [13,14]. Further investigation is important in order to establish which phytochemicals are connected with wound healing directly, and to elucidate their modes of action.

Although the therapeutic activity of *L. candidum* extract and its phytochemicals was demonstrated [74,75,76], their mechanism of action is still unknown.

In addition, pro-inflammatory cytokines IL-6 and IL-8 are associated with various diseases including ARDs, such as cancer, diabetes, cardiovascular diseases, multiple sclerosis, asthma, rheumatoid arthritis, and so on [7,8,9]. It is obvious that the stand-alone and combinational anti-inflammatory effects of kaempferol, citronellal, and humulene warrant further investigation. These results are in agreement with those obtained by Vachálková et al. [76], which show that some compounds of *L. candidum* (spirostanol saponins, two pyroline derivatives, jatropham and its glucoside, 2-fenylethyl-alpha-L-arabinopyranosyl-(1-->6)-beta-D-glucopyranoside, 2-phenylethylpalmitate, methylsuccinic acid and kaempferol) had significant anti-cancer properties [76].

With its rich collection of phytochemicals, *L. candidum* has promising therapeutic potential. Our results demonstrate a bright future for this plant and its compounds as prophylactic and therapeutic agents. However, additional clinical studies are warranted in order to establish the effectiveness of compounds from *L. candidum* in the treatment of chronic diseases.

## 4. Materials and Methods

### 4.1. Bioinformatic Assessment

For bioinformatical assessment, we selected the STITCH database found at http://stitch.embl.de/. This database contains most of the available information regarding proteins’ interaction with different chemicals. It can be searched according to the name of the chemical or its PubChem ID [63,64,65].

### 4.2. Preparation of Plant Material

Aerial parts of *L. candidum* were collected from the greenhouse at Ben-Gurion University of the Negev, Beer-Sheva, Israel. Leaves, flowers, and bulbs of *L. candidum* were dried by lyophilization and grounded for GC-MS analysis.

Ethanolic extracts were prepared from leaves and bulbs of *L. candidum* as described previously [27]. Plant tissues were homogenized, incubated at room temperature for 48 h in ethanol, centrifuged at 2000 rpm for 10 min, and the supernatant was evaporated by lyophilization. The pellet was dissolved in a minimal amount of 95% ethanol (0.5 mL) and diluted with water to a final concentration of 10 mg/mL.

### 4.3. GC-MS Analysis

Identification of volatile compounds is described in our previous publication [7].

### 4.4. Cell Cultures

All cell culture reagents including Dulbecco’s modified Eagle’s medium (DMEM), fetal bovine serum (FBS), L-glutamine, and antibiotics were purchased from Biological Industries (Kibbutz Beit Haemek, Israel).

Human pulmonary fibroblasts (HPFs), human dermal fibroblasts (HDFs), and 3T3-L1 adipocytes were propagated in DMEM supplemented with 10% FBS, 1% L-glutamine, and 1% antibiotic mixture, which included a combination of penicillin, streptomycin, and nystatin. The cells were grown in an incubator at 37 °C. The relative humidity was set at 95%, and the CO_2_ content was 5%.

### 4.5. Cytotoxicity Examination

Nontoxic concentrations of *Lilium candidum* extracts and compounds were determined by XTT assay. Succinctly, metabolically active cells reduce yellow salt, XTT (sodium 3’-[1-(phenylaminocarbonyl)-3,4-tetrazolium]-bis (4-methoxy6-nitro) benzene sulfonic acid hydrate), to form an orange formazan compound, as described in [7]. For measuring IL-6 and IL-8 levels, only non-toxic concentrations were used.

### 4.6. Measurement of Anti-Diabetic Activity

Ethanolic bulb and leaf extracts of *L. candidum* were tested for their anti-diabetic properties. The glucose uptake of the 3T3 adipocytes was determined as described previously [7,71]. Briefly, before the measurement, adipocytes were transferred to low-glucose serum-free media. After an overnight incubation, the cells were treated with bulb or leaf ethanol extracts of *L. candidum* for 1 h. For negative control, adipocytes were treated with the vehicle only. Insulin at a concentration of 100 nM was used as a positive control. After the 1-h incubation, the fluorescent glucose analog 2-(*N*-(7-nitrobenz-2-oxa1,3-diazol-4-yl) amino)-2-deoxyglucose (2-NBDG) was added for 30 min. The cells were then rinsed with PBS, and the fluorescence of intracellular 2-NBDG with excitation at 467 nm and emission at 538 nm was measured using a fluorescence microplate reader (POLARstar Omega, BMG LABTECH GmbH, Ortenberg, Germany).

### 4.7. Measurement of Interleukin Release

Senescent HPFs or HDFs were treated with specified concentrations of the extracts or compounds. After 3 days of exposure, we collected the medium and measured the concentrations of interleukin 6 and 8. The measurement was performed with R&D Systems ELISA kits according to the manufacturer’s instructions. For each experiment, standard curves were built, and the concentrations of interleukins were calculated.

### 4.8. Statistical Analysis

Experiments were repeated at least three times. All data were analyzed using Statistica, version 7, for Windows software (StatSoft, Inc., Tulsa, Oklahoma), and *p* < 0.05 was chosen as the minimal acceptable level of significance. Simple regression models were subsequently used to eliminate non-significant effects. Values are presented as means ± SD.

## Figures and Tables

**Table 1:** Medicinal properties of phytochemicals present in *Lilium candidum*.

Compound	Medicinal Uses	References
Kaempferol	Anti-apoptotic, pro-wound healing, anti-cancer, cardioprotective, anti-oxidant, pro-apoptotic, anti-allergic, anti-parasitic, anti-diabetic, anti-adipogenic, anti-thrombotic, anti-inflammatory, anti-metabolic syndrome, anti-bacterial, immunoregulatory, hepatoprotective, anti-atherosclerosis	[28,29,30,31,32,33,34,35]
Linalool	Anti-parasitic, anti-convulsant, anti-cancer, anti-bacterial, neuroprotective, anti-oxidant, anti-inflammatory, anti-Alzheimer, anxiolytic, hepatoprotective, anti-hyperalgesic, neuroprotective	[36,37,38,39,40,41,42,43]
Citronellal	Anti-fungal, insect repellant, hepatoprotective, anti-nociceptive, anti-inflammatory, anti-bacterial	[44,45,46]
Caryophyllene	Anti-cancer, anti-mutagenic, anti-bacterial, oxygen deprivation protective, neuroprotective, hepatoprotective, anti-convulsant, anti-diabetic, anti-microbial, anti-Alzheimer, pro-longevity, analgesic, nephroprotective	[47,48,49,50,51,52,53,54,55,56]
Humulene	Insecticidal, anti-cancer, anti-inflammatory	[51,57,58]
Neridiol	Anti-parasitic, antioxidant, neuroprotective, pro-wound healing, anti-microbial	[59,60,61,62]
